# Restoration of Endodontically Treated Molars Using All Ceramic Endocrowns

**DOI:** 10.1155/2013/210763

**Published:** 2013-12-22

**Authors:** Roopak Bose Carlos, Mohan Thomas Nainan, Shamina Pradhan, Roshni Sharma, Shiny Benjamin, Rajani Rose

**Affiliations:** ^1^Department of Conservative Dentistry and Endodontics, Vydehi Institute of Dental Sciences and Research Centre, No. 82, EPIP Area, Whitefield, Bangalore 560066, India; ^2^Dental Solutions, 157, 4th Main, BEML layout, Off ITPL Road, Thubarahalli, Bangalore 560066, India

## Abstract

Clinical success of endodontically treated posterior teeth is determined by the postendodontic restoration. Several options have been proposed to restore endodontically treated teeth. Endocrowns represent a conservative and esthetic restorative alternative to full coverage crowns. The preparation consists of a circular equigingival butt-joint margin and central retention cavity into the entire pulp chamber constructing both the crown and the core as a single unit. The case reports discussed here are moderately damaged endodontically treated molars restored using all ceramic endocrowns fabricated using two different systems, namely, CAD/CAM and pressed ceramic.

## 1. Introduction

Postendodontic restoration should preserve and protect the existing tooth structure, while restoring satisfactory esthetics, form, and function. The goal is to achieve minimally invasive preparations with maximal tissue conservation for restoring endodontically treated teeth. This will help to mechanically stabilize the tooth-restoration complex and increase surfaces available for adhesion.

A number of options are available in every clinical situation. The choice depends on the structural integrity of the tooth, esthetic, and protective requirements [1]. In this perspective, endocrowns can be considered as a feasible alternative to full crowns for restoration of nonvital posterior teeth, especially those with minimal crown height but sufficient tissue available for stable and durable adhesive cementation [2].

The evolution of ceramic technology especially dental CAD/CAM systems have enhanced the options to produce single all ceramic endocrowns with high biocompatibility and optimal mechanical properties [3].

In the present paper two ceramic endocrowns fabricated by different methods are presented as case reports.

## 2. Case 1

**Figure 1:**
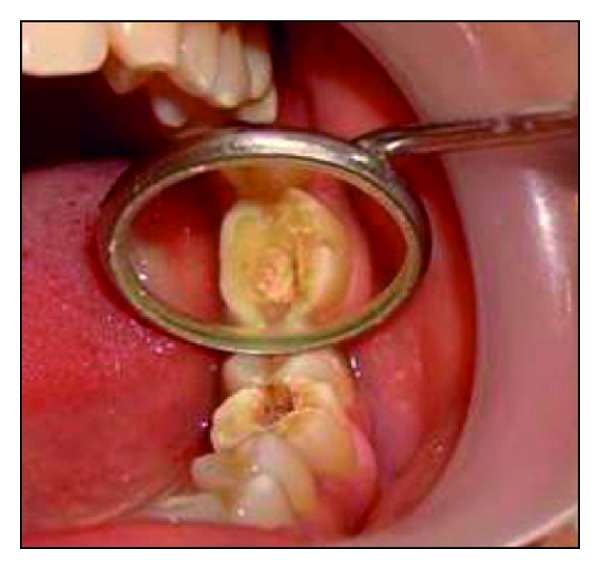
Postobturation occlusal view showing the amount of residual tooth structure.

A 32-year-old female patient reported for the filling of her lower 1st molar. On clinical examination tooth number 36 was root canal treated one month back (Figure 1). It was asymptomatic and the occlusogingival height of the remaining crown structure was approximately 4 mm. The radiographic findings revealed well obturated canals with no periapical changes.

A conservative approach of restoring the tooth with an endocrown was decided as the treatment option, as more than half the residual tooth structure was remaining and there were no occlusal wear facets. On additional request by the patient for an advanced and a prompt restoration, CAD/CAM ceramic was chosen.

**Figure 2:**
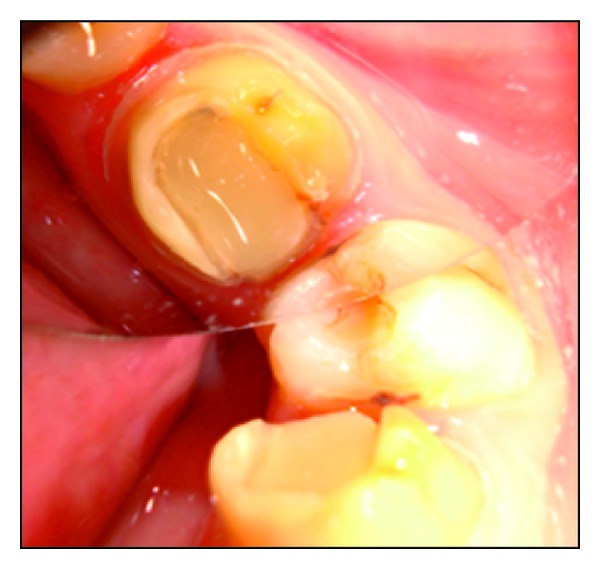
Tooth preparation for endocrown.

After removal of the provisional restoration, preparation for endocrown was initiated. Resin modified glass ionomer cement (Fuji II LC GC Corporation, Tokyo, Japan) was used to achieve a flat pulpal floor and to block the undercuts. The preparation consisted of a circular equigingival butt-joint margin and central retention cavity into the entire pulp chamber constructing both the crown and the core as a single unit. The appropriate reduction of the buccal and lingual walls was done (Figure 2).

Interocclusal space was carefully evaluated and occlusal reduction done to achieve a clearance of 2 mm. Shade-B_1_ was selected (VITAPAN Zahnfabrik, Germany). Retraction cord was placed and impressions made with polyvinyl siloxane impression material (Aquasil LV, Putty/Light Body, Dentsply DeTrey, Germany) using putty wash technique. Die stone model was fabricated.

**Figure 3:**
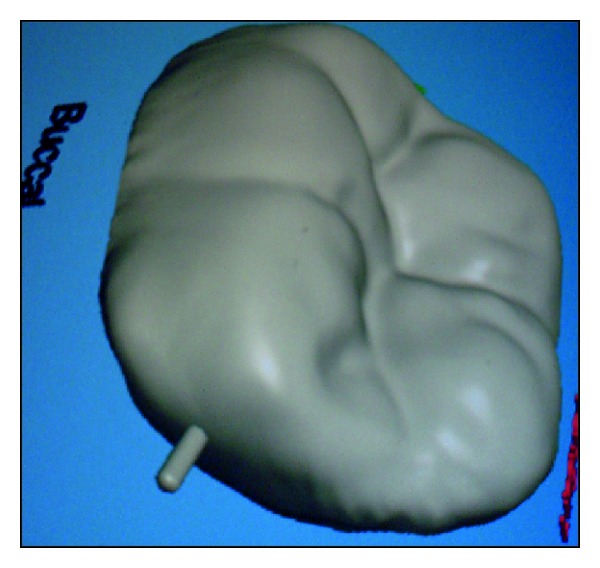
CAD/CAM image.

**Figure 4:**
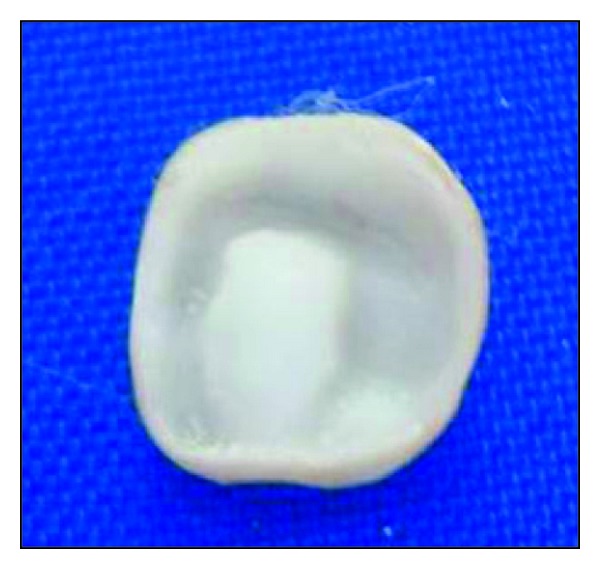
Tissue surface depicting the core and crown fabricated as a single unit.

*CAD/CAM Processing.* The three-dimensional reconstruction of the preparation was done using the Yenadent D40 milling machine (Yenadent, Istanbul, Turkey) and DWOS scanner (Dental Wings Inc., Montreal, Canada). The 3D scanning of the individual die and the antagonist arch for occlusal function (virtual articulation) were done. The milling was then initiated on a monolithic solid zirconia block (Metoxit AG, Thayngen, Switzerland) (Figures 3 and 4).

The finished endocrown was checked for shade, fit, and occlusion in the patient's mouth and then cemented using dual cure resin luting agent (Variolink, Ivoclar/Vivadent, Schaan/Liechtenstein).

**Figure 5:**
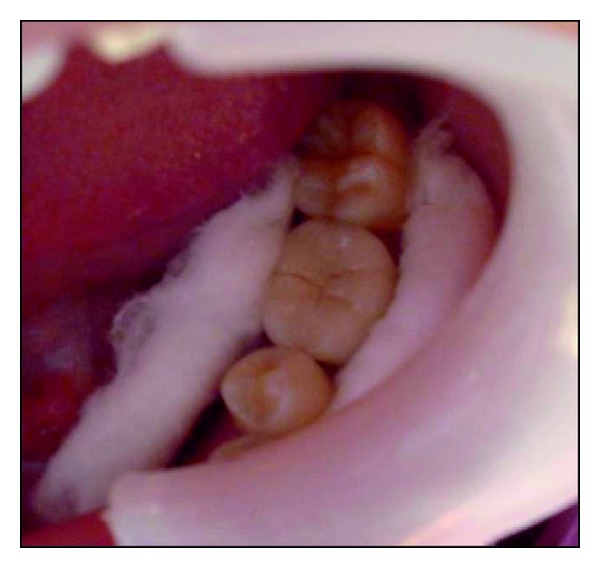
Occlusal view following final cementation.

**Figure 6:**
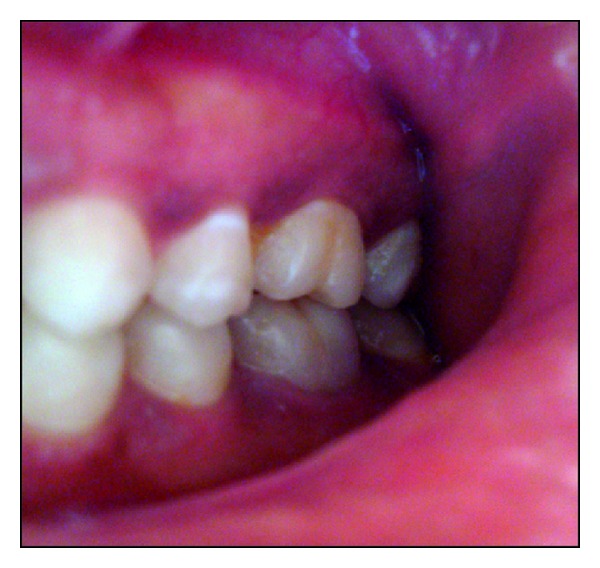
Buccal view of tooth 36 depicting the occlusion and imperceptible margins.

Clinical and radiographic evaluation was done and follow up after 28 months showed no secondary caries, fracture, discoloration or loosening/decementation of the crown (Figures 5 and 6).

## 3. Case 2

**Figure 7:**
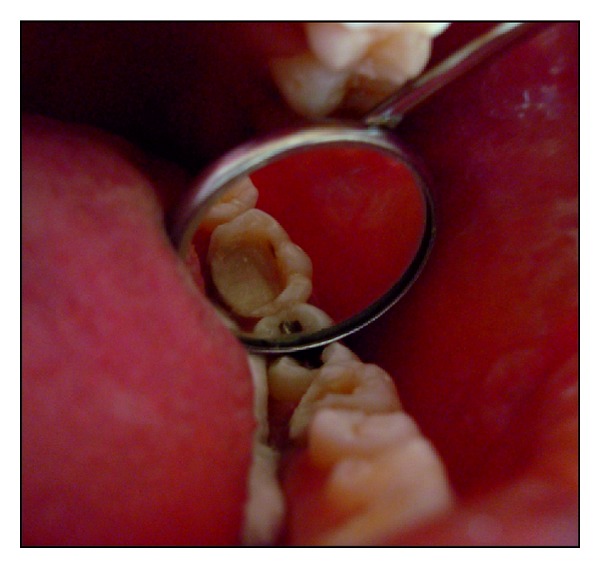
Occlusal view showing the amount of residual tooth structure postobturation.

**Figure 8:**
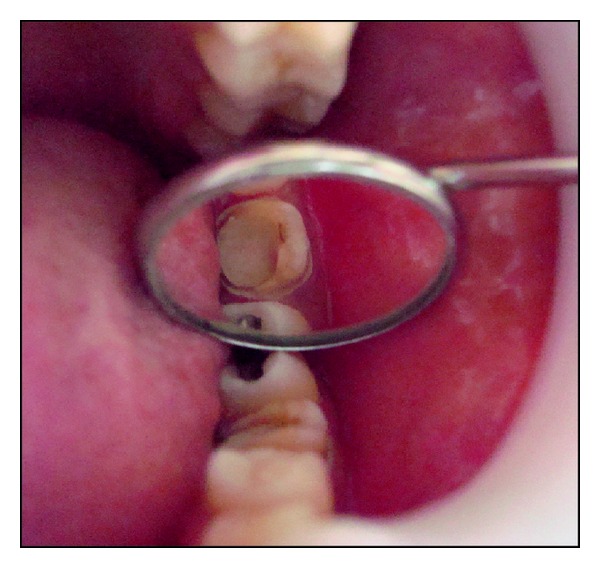
Tooth preparation for pressable ceramic endocrown.

**Figure 9:**
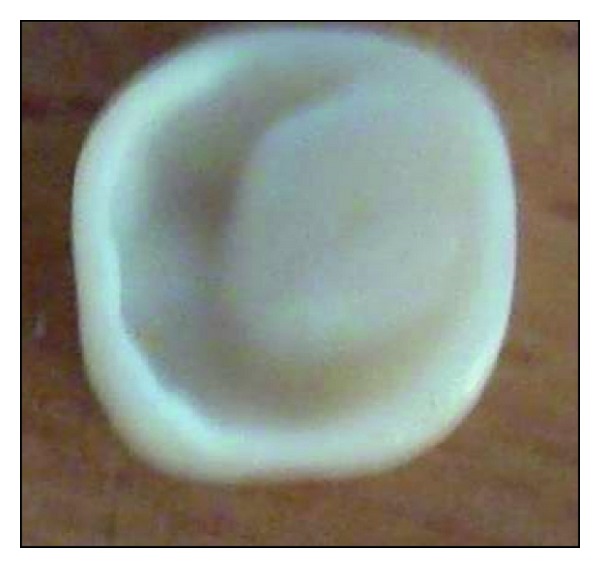
Tissue surface of pressed endocrown.

**Figure 10:**
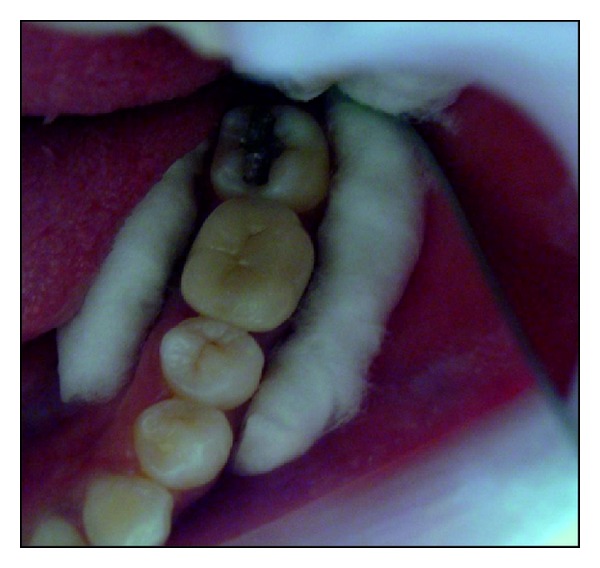
Occlusal view following final cementation.

**Figure 11:**
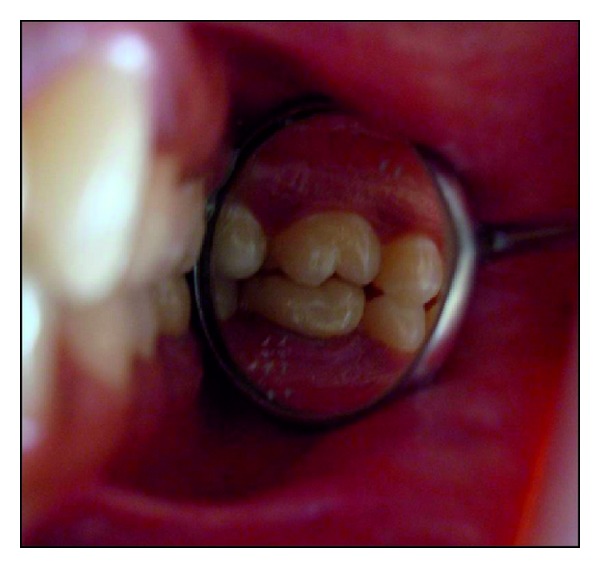
Buccal view of tooth 36 highlighting the excellent shade match and finish.

**Figure 12:**
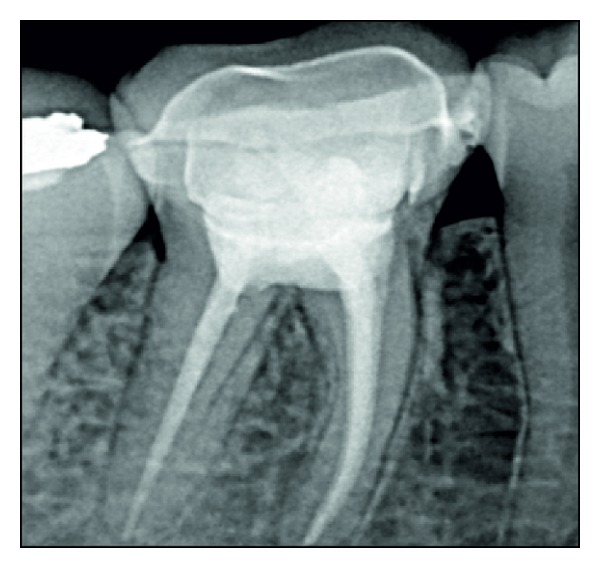
Radiographic view, postcementation. The supragingival finish line is clearly visible.

A 26-year-old female patient reported with a chief complaint of pain since 2 days. On radiographic examination radiolucency involving pulp of tooth 36 was seen. Based on the clinical and radiographic examination tooth 36 was diagnosed with acute irreversible pulpitis. Root canal treatment was performed. Based on the remaining tooth structure, that is, approximately 4-5 mm, occlusal evaluation, and patients esthetic demands, IPS E.max Press endocrown was decided as the treatment option. The endocrown preparation and the impression technique were performed as described in the previous case. IPS E.max Press HO Lithium-disilicate glass ceramic ingots (Ivoclar/Vivadent, Schaan/Liechtenstein) were used for the press technology. The restoration was fabricated according to the lost wax technique of investing and wax pattern burnout followed by pressing of the ceramic ingot in the pressable furnace at a press temperature of 915–920°C. It was then finished and polished with Proxyt pink polishing paste (Ivoclar/Vivadent, Schaan/Liechtenstein). The endocrown was cemented using a dual cure resin luting agent (Variolink, Ivoclar/Vivadent, Schaan/Liechtenstein). Clinical and radiographic evaluation was done and a 28-month followup showed no secondary caries, fracture, discoloration or loosening/decementation of the crown (Figures 7, 8, 9, 10, 11, and 12).

## 4. Discussion

A successful endodontic treatment has to be complemented with an appropriate postendodontic restoration to integrate the pulpless tooth with the masticatory apparatus [4]. When up to one half of the coronal tooth structure is missing, complete occlusal coverage is achieved conservatively using endocrown [5].

The concept of a conservative protective restoration for posterior endodontically treated teeth is not new. Amalcore, inlays, and onlays are based on this principle. The amalcore harnessed, the large and retentive contours of the root canal orifices, and the pulp chamber to provide a monoblock foundation. Inlays and onlays promoted the concept of a supragingival finish line and conservative preparations. The endocrown is an esthetic and conservative addition to this continuum.

All ceramic systems have gained popularity in recent times as they offer both esthetics and function [6]. The development of CAD/CAM systems and software offers several advantages in clinical practice. Custom shaping and precise milling of ceramic restorations is now a reality; furthermore, the adaptation of the inner surface of the restoration and the replication of the occlusal morphology are better. Restorations can be produced chairside and seated in one appointment. Inaccuracies are minimal and cross-contamination due to impression making and laboratory procedures is reduced. The net result is better patient compliance and satisfaction [6, 7].

On the other hand, pressable ceramic systems yield good functionality, retention, esthetics, and durability [2]. The main advantage of endocrown fabricated using the pressing method is the greater depth of the root extension and the option of using an articulator [3].

The 28-month followup of both types of endocrowns showed no esthetic and functional degradation. These results are in agreement with the previous studies [2, 8, 9].

Bindl and Mörmann demonstrated similar results in a clinical study of Cerec endocrowns cemented adhesively. 19 endocrowns were checked (4 premolars and 15 molars) in 13 patients over 28 months. Only one molar endocrown failed because of recurrent caries [9].

Similar results were reported by Lander and Dietschi where a three-year followup of two Empress II endocrowns showed satisfactory behavior in terms of esthetics, restoration stability, and tissue preservation [2].

Endocrowns have several advantages over conventional crowns like reduced number of interfaces in the restorative system. Stress concentration is less because of the reduction in the nonhomogenous material present [10, 11]. The preparation design is conservative compared to the traditional crown [5]. Involvement of the biological width is minimal [12]. In comparison to the post and core restorations, bonding surface offered by the pulpal chamber of the endocrown is often equal or even superior to that obtained from the bonding of a radicular post of 8 mm depth. The application and polymerization of resins is also better controlled [13].

As presented in the case reports, instead of modifying the existing tooth structure to suit the restorative needs, resin modified glass ionomer cement was used to block the undercuts, thereby further conserving sound tooth structure. The endocrown is luted with resin cement. The adhesive monoblock system achieved reduces the need for macroretentive geometry and provides more efficient outcome and better esthetics [7].

Endocrowns have their own disadvantages like, debonding and risk of root fracture because of the difference in the modulus of elasticity between the harder ceramic and softer dentin [3]. Hence case selection is critical for ensuring clinical success with endocrowns [14]. Endocrowns are indicated in cases where there are minimal functional and lateral stresses. When there is evidence of increased functional and lateral stresses as evident with steep occlusal anatomy, wear facets or parafunction, full coverage crown with or without post is the treatment of choice [12].

Based on current evidence, endocrowns fabricated using CAD/CAM and pressable ceramic technology can be considered as a reliable option for the restoration of moderately mutilated endodontically treated posterior teeth. However, long-term followup and longitudinal clinical studies are needed to ensure their overall success.
